# Monocyte chemoattractant protein-1 affects migration of hippocampal neural progenitors following status epilepticus in rats

**DOI:** 10.1186/1742-2094-10-11

**Published:** 2013-01-22

**Authors:** Yu-Wen Hung, Ming-Tsong Lai, Yi-Jhan Tseng, Chien-Chen Chou, Yung-Yang Lin

**Affiliations:** 1 Institute of Physiology, National Yang-Ming University, No.155, Sec. 2, Linong Street, Taipei, 112, Taiwan; 2 Institute of Brain Science, National Yang-Ming University, No.155, Sec. 2, Linong Street, Taipei, 112, Taiwan; 3 Institute of Clinical Medicine, National Yang-Ming University, No.155, Sec. 2, Linong Street, Taipei, 112, Taiwan; 4 Brain Research Center, National Yang-Ming University, No.155, Sec. 2, Linong Street, Taipei, 112, Taiwan; 5 Laboratory of Neurophysiology, Taipei Veterans General Hospital, No. 201, Sec. 2, Shih-Pai Rd, Taipei, 112, Taiwan; 6 Department of Neurology, Taipei Veterans General Hospital, No. 201, Sec. 2, Shih-Pai Rd, Taipei, 112, Taiwan

**Keywords:** Chemokine (C-C motif) receptor 2 (CCR2), Dentate gyrus, Epileptogenesis, Monocyte chemoattractant protein-1 (MCP-1), Neuroinflammation, Neuronal progenitor migration, Pilocarpine-induced status epilepticus, Rats

## Abstract

**Background:**

Epilepsy is a common brain disorder characterized by a chronic predisposition to generate spontaneous seizures. The mechanisms for epilepsy formation remain unknown. A growing body of evidence suggests the involvement of inflammatory processes in epileptogenesis. In the present study, we investigated the involvement of monocyte chemoattractant protein-1 (MCP-1) in aberrant migration of hippocampal progenitors in rats after the insult of status epilepticus (SE).

**Methods:**

SE was induced with pilocarpine in Sprague–Dawley rats. Transcriptional expression of MCP-1 in the dentate gyrus (DG) was measured using quantitative real-time PCR. From 1 to 28 days after SE, the temporal profiles of MCP-1 protein expression in DG were evaluated using enzyme-linked immunosorbent assay. Chemokine (C-C motif) receptor 2 (CCR2) expression in doublecortin-positive neuronal progenitors was examined using double-labeling immunohistochemistry. The involvement of MCP-1/CCR2 signaling in aberrant neuronal progenitor migration in the epileptic hippocampus was assessed in the SE rats using a CCR2 antagonist, RS102895, and the ectopic migration of neuronal progenitors was determined using Prox1/doublecortin double immunostaining.

**Results:**

After SE, MCP-1 gene was significantly upregulated and its corresponding protein expression in the DG was significantly increased on days 1 and 3. Some hilar ectopic progenitor cells of SE rats expressed the MCP-1 receptor, CCR2. Notably, the ectopic migration of neuronal progenitors into hilus was attenuated by a blockade of the MCP-1/CCR2 interaction with a selective CCR2 inhibitor, RS102895.

**Conclusions:**

An increase in dentate MCP-1 is associated with seizure-induced aberrant migration of neuronal progenitors through the interaction with CCR2. The upregulation of MCP-1 after an insult of SE may play a role in the generation of epilepsy.

## Background

Mesial temporal lobe epilepsy (MTLE) is a common type of epilepsy; more than half of patients with MTLE continue to experience seizures despite intensive medical treatment [1]. In some MTLE patients, a medical history-taking reveals an early precipitating brain insult and a following seizure-free latent period before the occurrence of unprovoked recurrent seizures in later childhood or adolescence. Epileptogenic insults may induce molecular and pathophysiological alterations in the hippocampus, including selective neuronal loss in the CA1, CA3 and dentate hilus, dentate neurogenesis, synaptic reorganization, gliosis and inflammatory responses [2-5]. These structural and functional changes in the hippocampal formation are potentially involved in the development of MTLE [2,3,6]. According to previous pathological studies in MTLE, the neuroplastic changes in the hippocampal dentate gyrus (DG) are important for local hyperexcitability and subsequent development of epilepsy [6]. However, the exact mechanisms underlying the epileptogenic structure alterations remain unclear.

Previous studies in experimental animals and human patients [5] have suggested a potential contribution of inflammatory process to seizure-related pathologies and the induction of epilepsy [7,8]. Seizures may trigger significant inflammatory responses, including activation of glial cells and secretion of inflammatory mediators by microglia, astrocytes and affected neurons [5]. Pathological alterations in animal models of epilepsy, such as neuronal cell death, reactive gliosis and neuroplastic changes, might be associated with these neuroinflammatory responses [9]. Therefore, neuroinflammation may participate in epileptogenesis [5,7-9].

Several chemokines and chemokine receptors expressed in normal brain participate in various physiological processes, including the regulation of neuronal development and migration, synaptic activity and cellular communication [10,11]. These molecules can be up-regulated in response to inflammatory brain insults [11-13]. The chemokine monocyte chemoattractant protein-1 (MCP-1) is increased in patients with intractable epilepsy, suggesting its possible involvement in the pathogenesis of epilepsy [14-16]. In animal studies, MCP-1 expression in the hippocampus was up-regulated after acute seizures [17,18]. MCP-1, one of the most commonly expressed chemokines in the inflamed brain [19], belongs to a member of the cysteine-cysteine (CC) chemokine gene family. Members of this gene family attract monocytes and activate T cells and B cells through their interaction with the seven-transmembrane domain, G protein-coupled receptors chemokine (C-C motif) receptor 2 (CCR2) or CCR9 [20]. Elevated MCP-1 expression in the inflamed brain is related to the activation and recruitment of macrophages/microglia and granulocytes near the site of injury [18] and is also related to the breakdown of the blood–brain barrier [19,21]. Accordingly, MCP-1 in the epileptic brain might participate in the inflammation-related epileptogenesis of MTLE.

In addition to involving inflammatory response, MCP-1 may enhance the migration of adult neural progenitors *in vitro*[22,23]. MCP-1 may induce neural progenitor migration from the subventricular zone toward the damaged brain regions after focal ischemia, since expression of MCP-1 receptor CCR2 has been identified in the migrating neuroblasts [23]. Also, MCP-1 is important in attracting neural progenitors toward sites of neuroinflammation in the hippocampus [13,24]. Thus, MCP-1 is a potential regulator of neuronal progenitor migration in response to brain insults [23]. In the epileptic hippocampus, ectopic granule cells derived from newborn neurons were observed in the dentate hilus following seizure activity [25,26]. The aberrant migration of neuronal progenitors may contribute to the formation of epileptic neuronal circuits and hyperexcitability in the hippocampus [25-27]. In response to these findings, we hypothesized that MCP-1 participates in epileptogenesis through its attraction of neural progenitors in the subgranular zone (SGZ) to form hilar ectopic granule cells.

To study the pro-epileptogenic role of MCP-1 in the seizure-induced aberrant reorganization of the dentate hilus, we evaluated the temporal profiles of MCP-1 expression in the DG after pilocarpine-induced status epilepticus (SE) and determined whether MCP-1 enhances the migration of adult neural progenitors towards ectopic locations.

## Methods

### Animals

Adult male Sprague–Dawley rats weighing 200 to 350 g were housed in a controlled environment (lights on from 8:00 A.M. to 8:00 P.M.) and given free access to food and water. Animal protocols were approved by the Taipei Veterans General Hospital Institutional Animal Care and Use Committee (Permit Number: 97–100), Taipei, Taiwan, and conformed to the National Institutes of Health (NIH) Guidelines of USA for the care and use of laboratory animals.

### Pilocarpine-induced status epilepticus

In order to assess MCP-1 gene expression profiles of hippocampal DG in response to a SE insult, pilocarpine hydrochloride (325 mg/kg; Sigma, St. Louis, MO, USA) was intraperitoneally (i.p.) administered in 6 rats, while normal saline of the same volume was i.p. injected in 7 control rats. In a separate experiment for the analysis of protein and histopathological alterations, rats were pretreated with scopolamine methylnitrate (1 mg/kg, i.p.; Sigma) to reduce peripheral cholinergic side effects [28]. Thirty minutes later, pilocarpine hydrochloride was given to induce SE. In age-matched control rats, pilocarpine was replaced with normal saline. Animal behavior was monitored, and only the rats exhibiting convulsive SE at stage 5 of Racine’s scale were enrolled for subsequent experimental manipulations [25]. Two hours after SE onset, rats were treated with diazepam (10 mg/kg, i.p.; China Chemical & Pharmaceutical Co., Ltd., Taipei, Taiwan) to terminate seizures.

### Administration of RS102895, an antagonist of CCR2b

As a potent and specific antagonist of CCR2b, RS102895 (Tocris Bioscience, Ellisville, MO, USA) can inhibit MCP-1/CCR2 signaling in rodents [29,30]. One day after SE induction, SE rats were subcutaneously treated with 10 mg/kg of RS102895 (in a solution of 10% dimethyl sulfoxide (DMSO; Sigma) and 90% normal saline) for 3 days. Then the dose of RS102895 was 5 mg/kg for subsequent days until one day prior to sacrifice.

### Preparation of the DG

Animals were deeply anesthetized via isoflurane inhalation (AErrane, Baxter, Guayama, PR, USA) and decapitated. The brain was quickly removed from the skull and placed into ice-cold phosphate-buffered saline (PBS). Hippocampal DG tissue was carefully dissected out from each cerebral hemisphere by sliding 30-gauge needles superficially along the boundaries of the DG and Ammon’s horn within the hippocampus [31]. The left or right DG was chosen at random for total RNA isolation and protein extraction.

### RNA isolation and quantitative real-time reverse transcriptase PCR (qRT-PCR)

To obtain hippocampal dentate RNA for qRT-PCR, SE rats were sacrificed 30 min after diazepam injection. Total RNA was isolated using TRIzol reagent (Invitrogen, Carlsbad, CA, USA) according to the manufacturer’s instructions. The integrity of each RNA sample was confirmed using agarose gel electrophoresis and an Agilent 2100 Bioanalyzer (Agilent Technologies, Santa Clara, CA, USA).

qRT-PCR was performed in triplicate to validate the expression of MCP-1 [32]. First, total RNA was reverse-transcribed into cDNA. A 20 μL reaction volume containing 2.5 μg of total RNA was used in the first-strand cDNA synthesis reaction. Oligo dT (0.5 μg) and dNTP mix (1 μL, 10 mM) were added to total RNA. The mixture was heated to 65°C for 5 min and quickly chilled on ice for 2 min. Then, 5× first-strand buffer (5 μL), dTT (1 μL) and SuperScript III reverse transcriptase (1 μL, 200U, Invitrogen) were added to the reverse transcription reaction mixture. Following incubation of the mixture at 42°C for 60 min and then 70°C for 15 min, first-strand cDNA was generated. Next, qRT-PCR was carried out using the SYBR Green chemistry for amplicon detection on the LightCycler 480 Real-Time PCR System (Roche Applied Science, Indianapolis, IN, USA). Each sample for qRT-PCR contained the following: 10 μL 2× FastStart Universal Probe Master (Roche Applied Science), 1 μL primers for MCP-1 (forward: TGAACTTGACCCATAAATCTGAAG, reverse: AAGGCATCACATTCCAAATCAC) and for GAPDH (forward: CTCCCATTCTTCCACCTTTG, reverse: CTTGCTCTCAGTATCCTTGC), and 1 μL cDNA. The final volume was adjusted to 20 μL with the addition of H_2_O. Temperature cycling was performed as follows: 3 min polymerase activation at 95°C followed by 45 cycles of denaturation at 95°C for 30 sec, annealing at 55°C for 30 sec and extension at 72°C for 30 sec. The fluorescence signal was read during the extension step of each cycle. To distinguish between the specific and non-specific products and primer dimers, melting curves were obtained by ramping the temperature 1°C every second from 55°C to 95°C after PCR amplification. The relative fold change of gene expression was calculated by the value of 2^∆C_T_ (compared to the control group) and normalized to the expression of the housekeeping gene GAPDH.

### Detection of MCP-1 protein in the DG by enzyme-linked immunosorbent assay (ELISA)

DG tissue was dissected at 1, 3, 7, 14 and 28 days after SE in SE rats (n = 6 per time points) and at 1 day after saline treatment in control rats (n = 6). Total protein was extracted using T-PER tissue protein extraction reagent (Pierce Biotechnology, Inc., Rockford, IL, USA) containing EDTA-free Complete protease inhibitors (Roche Applied Science), according to the manufacturer’s recommendations. Total protein concentration was determined using the Bio-Rad protein assay kit (Bio-Rad, Hercules, CA, USA). MCP-1 protein concentration from each sample was evaluated using the BD OptEIA Rat MCP-1 ELISA kit (BD Biosciences Pharmingen, San Diego, CA, USA), according to the manufacturer’s instructions, and then normalized to the total protein content. The data of MCP-1 protein concentration were presented as pg/μg of total protein.

### Tissue preparation and immunohistochemistry

Rats were deeply anesthetized with Zoletil 50 anesthesia (10 mg/kg, i.p.; Virbac Laboratories, Carros, France) and perfused transcardially with ice-cold normal saline, followed by 4% paraformaldehyde in PBS (10 mM, pH 7.4). Brain tissue was removed immediately after perfusion, post-fixed in 4% paraformaldehyde overnight at 4°C and cryoprotected with 30% (w/v) sucrose in PBS. Coronal sections (40 μm) through the dorsal hippocampus (AP = −1.8 to −6.12 mm) were prepared using a freezing microtome (CM1900, Leica, Heidelberg, Germany) and stored in an antifreeze solution (50 mM phosphate buffer, 15% glucose, 30% (v/v) ethylene glycol, 0.05% sodium azide; pH 7.4) at −20°C until further testing [33]. Every 12^th^ section (480 μm apart) through the hippocampus was selected in each rat.

To detect the expression patterns of MCP-1 and prospero homeobox protein 1 (Prox1), free-floating tissue sections were incubated in 3% H_2_O_2_ for 30 min to block endogenous peroxidase. After a PBS rinse for 10 min, sections were placed in 3% NHS at room temperature (RT) for 30 min. The sections were then incubated overnight at 4°C with goat polyclonal MCP-1 primary antibody (1:100; sc-1785, Santa Cruz Biotechnology, Santa Cruz, CA, USA) and rabbit polyclonal Prox1 primary antibody (1:2000; ab5475, Millipore, Temecula, CA, USA). Sections were then washed and incubated for 1 hr at RT in biotinylated-secondary anti-goat antibody (1:300; Vector Laboratories, Burlingame, CA, USA) and anti-rabbit antibody (1:300; Vector Laboratories), respectively. After washing three times, the sections were incubated in the avidin-biotin complex (Vector Laboratories) for 30 min. The sections were then washed three times and reacted with diaminobenzidine tetrahydrochloride (DAB; Sigma) (in 1.5% NaCl containing 0.015% hydrogen peroxide) for 5 min. The color reaction was stopped with ice-cold PBS.

To characterize the cellular source of MCP-1, tissue sections were blocked in 3% normal horse serum (NHS) (in 0.1 M Tris, 0.1% Triton X-100) for 30 min at RT and then incubated overnight at 4°C with a goat polyclonal MCP-1 primary antibody (1:100; sc-1785, Santa Cruz Biotechnology). Following primary antibody treatment, sections were washed in PBS, incubated with DyLight 594-conjugated secondary anti-goat antibody (1:500; Jackson ImmunoResearch Laboratories, West Grove, PA, USA) for 1 hr at RT, washed in PBS and incubated in mouse monoclonal primary antibodies as follows: CD11b antibody (1:100; ab1211, Abcam, Cambridge, MA, USA), neuronal neuclei protein (NeuN) antibody (1:500; MAB377, Millipore), and glial fibrillary acidic protein (GFAP) antibody (1:1000; MAB360, Millipore) at 4°C overnight. After washing in PBS, sections were incubated in DyLight 488-conjugated secondary anti-mouse antibody (1:500; Jackson ImmunoResearch Laboratories) for 1 hr at RT and then washed in PBS.

To determine whether neuronal progenitors in DG express MCP-1 receptor CCR2, immunofluorescent double staining was performed. The tissue sections were first blocked in 3% NHS for 30 min at RT and then incubated with rabbit polyclonal CCR2 antibody (1:100; ab21667, Abcam) for 48 hr at 4°C. Following primary antibody treatment, sections were washed in PBS, incubated with DyLight 488-conjugated secondary anti-rabbit antibody (1:500; Jackson ImmunoResearch Laboratories) for 1 hr at RT, and washed in PBS. Negative controls for CCR2 immunochemistry were performed by blocking with the CCR2 peptide (ab22407, Abcam) that was used as an immunogen for the CCR2 antibody production and also by omitting the primary CCR2 antibody. The tissue sections were then incubated with a goat polyclonal doublecortin (Dcx) antibody (1:100; sc-8066, Santa Cruz Biotechnology) for 48 hr at 4°C. After washing in PBS, sections were incubated in DyLight 594-conjugated secondary anti-goat antibody (1:500; Jackson ImmunoResearch Laboratories) for 1 hr at RT and then washed in PBS.

Double-immunofluorescence labeling for Prox1 and CCR2 was performed using 1:200 mouse monoclonal Prox1 antibody (MAB5652, Millipore) and 1:500 DyLight 549-conjugated secondary anti-mouse antibody (Jackson ImmunoResearch Laboratories), followed by rabbit polyclonal CCR2 antibody (1:100; ab21667, Abcam) and DyLight 488-conjugated secondary anti-rabbit antibody (1:500; Jackson ImmunoResearch Laboratories). To ensure that the hilar ectopic Prox1-expressing cells are neuronal progenitors, double-immunofluorescence staining for Prox1 and Dcx was performed. The sections were incubated in a rabbit polyclonal Prox1 antibody (1:1000; ab5475, Millipore) and DyLight 488 -conjugated secondary anti-rabbit antibody (1:500; Jackson ImmunoResearch Laboratories), followed by a goat polyclonal Dcx antibody (1:100; sc-8066, Santa Cruz Biotechnology) and DyLight 594-conjugated secondary anti-goat antibody (1:500; Jackson ImmunoResearch Laboratories).

All of the tissue sections were then mounted on gelatin-coated slides and air-dried. The sections for immunoperoxidase staining were coverslipped with DPX Mountant (Fluka, Buchs, Switzerland), and those for immunofluorescent staining were coverslipped with Vectashield HardSet mounting medium with DAPI (H-1500; Vector Laboratories). Immunoperoxidase-stained sections were detected using a bright-field microscope (Olympus BX61, Olympus Optical Co. Ltd.) and photomicrographs were captured using a digital camera (Olympus CC12, Olympus Optical Co. Ltd.) with a 10× objective.

To confirm the types of MCP-1 expressing cells, fluorescent images were detected using an epi-fluorescent microscope (Olympus BX61, Olympus Optical Co. Ltd.) and photomicrographs were captured using a digital camera (Olympus CC12, Olympus Optical Co. Ltd.) with a 10× objective. In addition, the hilar ectopic Dcx/CCR2-expressing neuroblasts, Prox1/CCR2-expressing cells and Prox1/Dcx-expressing progenitors, were verified by a laser scanning confocal microscope (Olympus FV1000, Olympus Optical Co. Ltd.) with 20× and 60× objectives.

### Stereology of Prox1/Dcx double-labeled cells

The number of Prox1/Dcx-positive cells in the bilateral dentate hilus was estimated in every 12^th^ section using the optical fractionator [34] with StereoInvestigator 9 software (Microbrightfield, Williston, VT, USA). The sections were viewed in an epi-fluorescent microscope (Olympus BX51, Olympus Optical Co. Ltd.) with a motorized stage. The contours around regions of interest were traced with a 10× objective and the Prox1/Dcx double-labeled cells were counted with a 40× objective. The hilus was demarcated by the border of the upper and lower blades of the granule cell layer, and a straight line drawn between the two lateral tips of granule cell layer. Because it was difficult to determine the border between CA3c and hilus in Prox1/Dcx immunostained sections, the immunoreactive cells in CA3c were included in this study.

**Figure 1:**
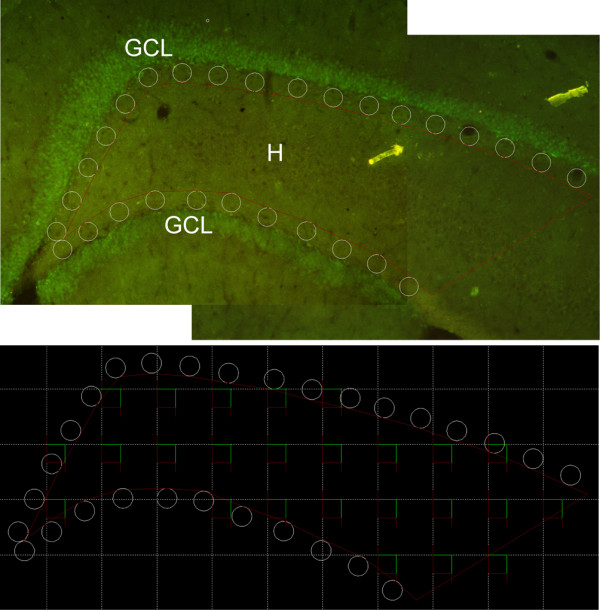
**Schematic illustration of the stereological methods for quantification of Prox1/Dcx-positive cells in hilus.** The upper panel represents the tracing procedure for the contour of the hilar region. Some 50 μm diameter circles were placed on the interior of the granule cell layer to exclude the SGZ. The boundaries of hilus was traced along the edge of the circles in hilus, from the vertex of the granule cell layer to a straight line drawn between the two lateral tips of the granule cell layer. The lower panel demonstrates a counting frame of 50×50 μm distributed in a randomly oriented 150×150 μm XY grid. Only the grids within the traced contour were included. GCL: granule cell layer; H: hilus.

To exclude the Prox1/Dcx-positive cells at the border of the granule cell layer and hilus, 50 μm diameter circles were placed on the interior of the granule cell layer and the contour of the hilus was traced along the edge of these circles in hilus, from the vertex of the granule cell layer to a straight line drawn between the two lateral tips of granule cell layer [35]. Within the contour of the hilus, a counting frame of 50×50 μm was distributed in a randomly oriented 150×150 μm XY grid (Figure 1). The Prox1/Dcx double-labeled cells at each counting frame were counted within the optical dissector height (20 μm) centered between top and bottom guard zones (5 μm) to estimate the number of hilar ectopic neuronal progenitors. In each section, the cell count and total volume of traced hilar region were estimated according to the formula developed by West *et al.*[34,36] and Cavalieri’s principle [37], as described previously [35,38]. The data were expressed as the number of Prox1/Dcx-positive cell per unit volume of the hilus (cells/mm^3^ + SEM).

### Statistical analysis

The qRT-PCR data were statistically analyzed by independent-samples *t*-test. MCP-1 protein concentrations in the DG at the indicated time points in the SE groups were compared with those in the control group using one-way ANOVA followed by Dunnett’s *post hoc* test. The density of hilar ectopic Prox1/Dcx-positive cells in all groups was statistically analyzed using one-way ANOVA followed by the Scheffe *post hoc* test. The data were shown as the mean + SEM. The significance level was set at *P* <0.05.

## Results

### MCP-1 expression in the hippocampal DG after SE

**Figure 2:**
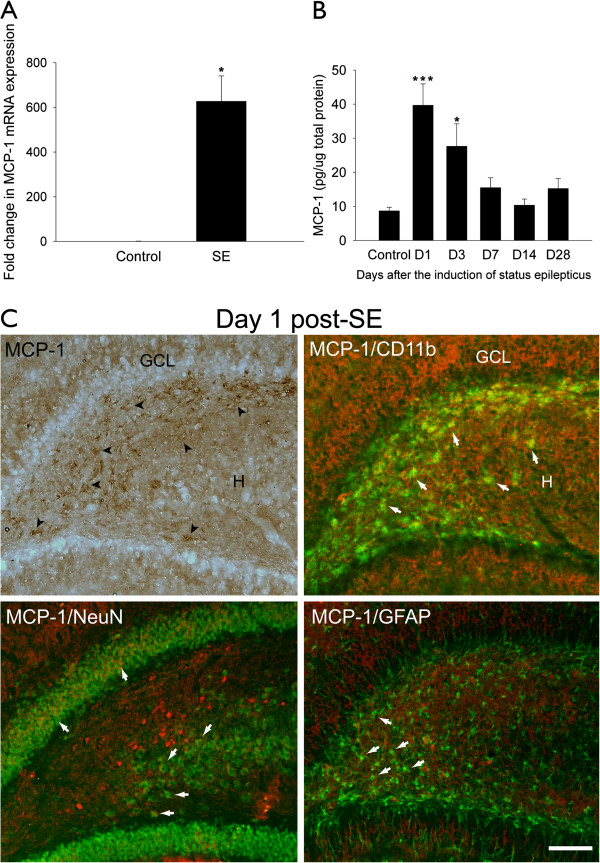
**Upregulated MCP-1 expression in the DG after SE.** (**A**) Mean fold-change (+ SEM) of MCP-1 gene expression in SE rats with respect to control rats. ^*^P <0.05 compared with the control group. (**B**) Mean concentration (+ SEM) of MCP-1 protein at 1 to 28 days after SE. ^*^P <0.05, ^***^P <0.001 compared with the control group. (**C**) Distribution patterns in the DG of MCP-1 expression (left upper panel) and its co-labeling with CD11b (right upper panel), NeuN (left lower panel) and GFAP (right lower panel) on day 1 after SE. Arrowheads denote MCP-1-positive cells in hilus. Arrows denote MCP-1-positive cells (red) co-labeled with CD11b, GFAP and NeuN (green), respectively. Scale bar: 100 μm. GCL: granule cell layer; H: hilus.

There was a significant increase in MCP-1 gene expression in response to seizure insults compared with the control (*P* = 0.022; independent-samples *t*-test; n = 6 to 7 rats per group) (Figure 2A). Compared with the control levels, MCP-1 protein expression in the DG was significantly increased one day (*P* = 0.000052) and three days (*P* = 0.013) post-SE (one-way ANOVA, F(df = 5, 30) = 8.17, *P* = 0.000058, Dunnett’s *post hoc* test; n = 6 rats per time point) (Figure 2B). Double-labeling immunohistochemical analysis at one day after SE showed that MCP-1 expression was mainly found in CD11b-positive reactive microglia within the hilar area and that some MCP-1 expressing cells were co-labeled with GFAP and NeuN (Figure 2C).

### Distribution of MCP-1 receptor CCR2 in the hippocampal DG after SE

**Figure 3:**
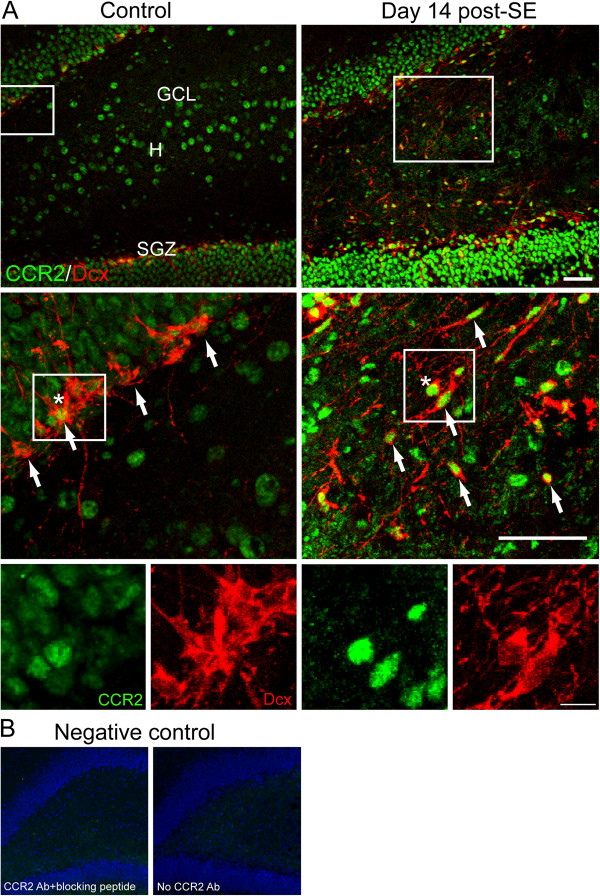
**Confocal micrographs of MCP-1 receptor, CCR2 and Dcx in the hippocampal DG.** (**A**) As shown in top panels, CCR2-positive cells (green) appeared in the GCL, SGZ and hilus. Within the SGZ, CCR2-positive cells were double-labeled with a neuroblast marker Dcx (red). Note that Dcx-positive cells appeared ectopically within the hilus after SE, and a subset of these cells expressed CCR2. The middle and bottom panels show projected z-stack confocal images to demonstrate colocalization of CCR2 with Dcx. White brackets mark the magnified views of the area. Arrows denote CCR2/Dcx-positive cells. The cell denoted by the asterisk is enlarged to illustrate the pattern of CCR2 and Dcx labeling. Scale bar: top and middle, 50 μm; bottom, 10 μm. GCL: granule cell layer; H: hilus; SGZ: subgranular zone. (**B**) Negative control immunostaining images for CCR2 when the CCR2 primary antibody was omitted (right) or eliminated by a blocking peptide (left). Neuclei were counterstained with DAPI (blue).

In the DG, CCR2-positive cells were found in the hilus, the SGZ and the granule cell layer. In the SGZ, CCR2-expressing cells were double-labeled with Dcx in control and SE rats, indicating that neural progenitors express CCR2. Furthermore, we also found the ectopically located Dcx-positive cells in the hilar region of SE rats, and a subset of these cells also expressed CCR2 (Figure 3A). CCR2 immunostaining was abolished either by pretreatment with a synthetic immunogenic peptide or by omitting the primary antibody (Figure 3B).

### Effect of a CCR2 antagonist RS102895 on the ectopic migration of neuronal progenitors after SE

**Figure 4:**
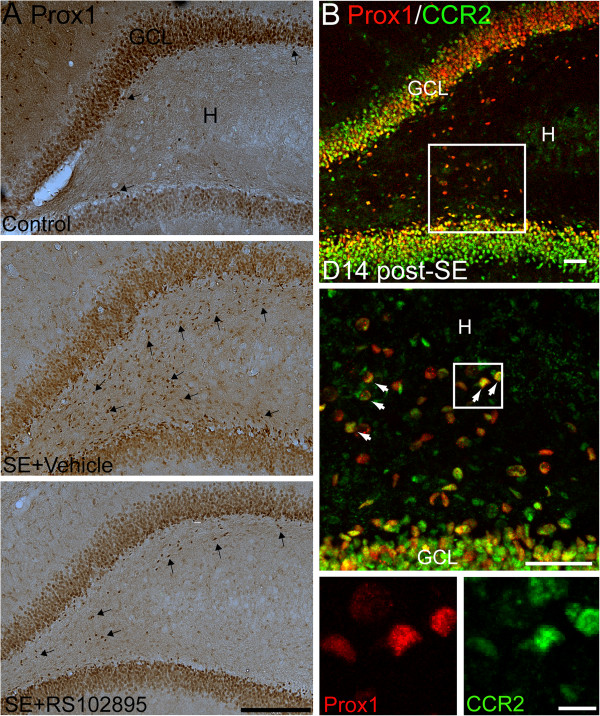
**Effects of MCP-1/CCR2 inhibition on the formation of hilar-ectopic granule cells.** (**A**) Granule cells were immunolabeled with the granule cell-specific marker Prox1 in controls, vehicle-treated SE rats, and RS102895-treated SE rats. In SE rats, Prox1 expression was observed not only in the granule cell layer but also in the hilus. Note that the number of Prox1-expressing cells in the hilus was reduced in the SE rats treated with RS102895. Arrows denote the Prox1-positive cells. Scale bar: 200 μm. (**B**) Confocal micrographs for Prox-1 (red) and CCR2 (green) showing a co-expression of CCR2 in Prox-1-expressing cells in the hilus at 14 days post-SE. The middle and bottom panels show projected z-stack confocal images to demonstrate colocalization of Prox1 with CCR2, as denoted by arrows. White brackets mark the magnified views of the area. Scale bar: top and middle, 50 μm; bottom, 10 μm. GCL: granule cell layer; H: hilus.

**Figure 5:**
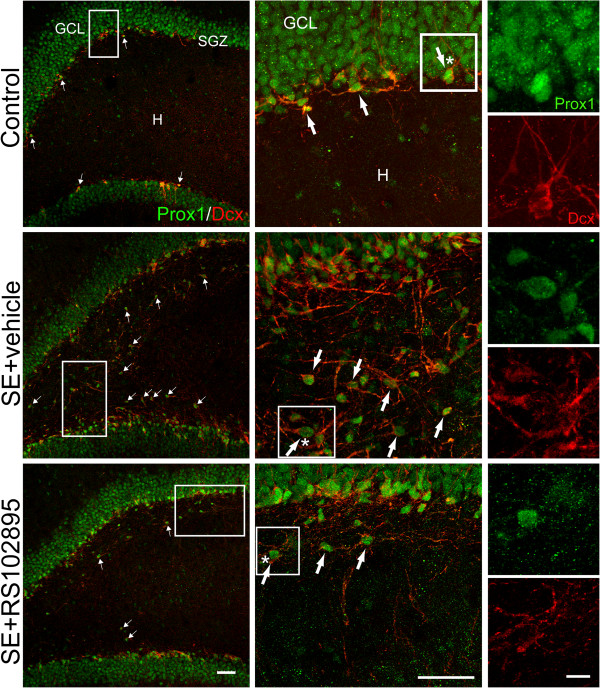
**Confocal micrographs of Prox1-expressing progenitors (green) co-labeled with Dcx (red) in the hilus regions of control rats, vehicle-treated SE rats, and RS102895-treated SE rats on Day 14 after SE.** The middle and right panels show projected z-stack confocal images to demonstrate colocalization of Prox1 with Dcx. Prox1/Dcx-expressing progenitors appeared within the SGZ in control and SE rats. Note that Prox1/Dcx-expressing progenitors appeared ectopically within the hilus after SE, and the formation of these hilar ectopic neuronal progenitors was attenuated by administration of RS102895. Arrows denote the Prox1/Dcx-positive cells. White brackets mark the magnified views of the area. The cell denoted by the asterisk is enlarged to illustrate the pattern of Prox1 and Dcx labeling. Scale bar: left and middle, 50 μm; right, 10 μm. GCL: granule cell layer; H: hilus; SGZ: subgranular zone.

As shown in Figure 4A, Prox1 expression in the controls was found throughout the granule cell layer. In the SE rats, Prox1-expressing cells in the hilus at 14 days post-SE also expressed CCR2 (Figure 4B). According to double-immunofluorescence staining for Prox1 and Dcx, Prox1/Dcx-expressing neuronal progenitors were found primarily within the SGZ in controls. In SE rats, the number of Prox1/Dcx-expressing neuronal progenitors in the hilus was decreased after administration of a CCR2 antagonist RS102895 (Figure 5).

**Figure 6:**
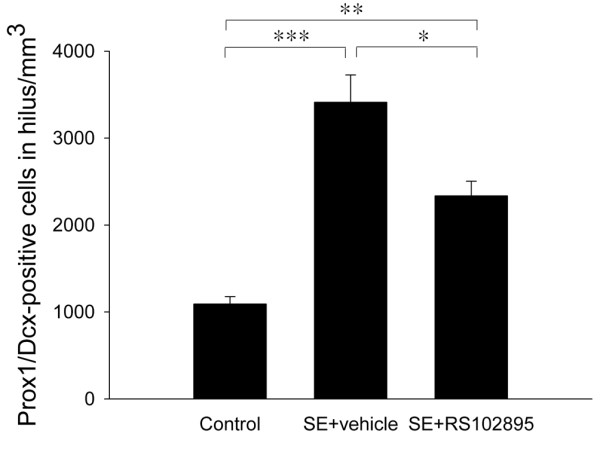
**Mean density (+ SEM) of Prox1/Dcx-positive cells in the dentate hilus of controls and SE rats on day 14 after SE.** The number of Prox1/Dcx-positive cells was decreased in RS102895- treated SE rats compared with vehicle-treated SE rats. ^*^P <0.05, ^**^P <0.01, ^***^P <0.001.

Quantitative stereological analysis further confirmed that the density of Prox1/Dcx-expressing neuronal progenitors in the hilus was significantly higher in the SE rats treated with vehicle compared with control rats (*P* = 0.0001; one-way ANOVA, F(df = 2, 9), *P* = 0.00010, Scheffe *post hoc* test; n = 5 sections per rat, 4 rats per group) and the SE rats treated with RS102895 (*P* = 0.018; one-way ANOVA, F(df = 2, 9), *P* = 0.00010, Scheffe *post hoc* test; n = 5 sections per rat, 4 rats per group) (Figure 6). Table 1 shows a summary of the estimation of Prox1/Dcx-expressing cell density in the hilus.

**Table 1:** Estimates of Prox1/Dcx-positive cells in the hilus

	**Control**	**SE-vehicle**	**SE-RS102895**
Cells counted/rat^a^	9.25 ±0.48	28.50 ±5.11	20.25 ±1.03
Estimated cell number^a^	1726.26 ±87.51	5498.83 ±917.25	3916.08 ±294.36
Total area (mm^2^)^a^	3.85 ±0.21	3.99 ±0.35	4.00 ±0.18
Mean CE^b^	0.13	0.08	0.09

CE is coefficient of error.

^a^Values are mean ± SEM.

^b^Mean CE is calculated as √Mean CE^2^[34].

## Discussion

Following SE, MCP-1 expression was up-regulated in the DG and CCR2-exhibiting neuroblasts appeared ectopically in the hilus. Notably, the ectopic migration of neuronal progenitors into hilus was significantly decreased by inhibiting the MCP-1/CCR2 interaction. These findings suggest an important role of MCP-1 in the aberrant migration of neuronal progenitors after SE.

### Abnormalities in DG and epileptogenesis

The pathogenic mechanisms of MTLE remain largely unknown. As a gating structure in the hippocampal circuit [6], the DG has been an important target in human and experimental studies. Mossy fiber sprouting, selective neuron loss and abnormal neurogenesis have been considered to be histopathological changes contributing to synaptic reorganization and local hyperexcitability in the DG [6]. Several recent studies have implicated aberrant neurogenesis in the DG in the development of epilepsy. Acute seizure activity promotes the rate of dentate neurogenesis [4,39,40], and prolonged seizures may dysregulate the migration of the DG progenitor cells and lead to hilar-ectopic granule cells [25,26,41,42]. Consistent with previous research, the present study found ectopic Dcx-positive neuroblasts and Prox1-positive granule cells in the hilus after SE induction (Figures 3, 4, 5). These ectopic and pre-existing granule neurons may integrate abnormally with each other and contribute to network hyperexcitability [25].

### MCP-1 related neuroinflammation in epilepsy

The interaction of MCP-1 and its receptor CCR2 in the brain can mediate monocyte infiltration and microglial recruitment [43] and contribute to tissue injury via the modulation of blood–brain barrier permeability [18,19] under neuroinflammatory conditions. Increased MCP-1 expression has been detected in rodent brains after acute seizures [17,18,44] and in pathological brain tissue of epilepsy patients [14-16]. In the present study, MCP-1 expression in the DG was upregulated significantly by pilocarpine-induced SE, and this change persisted for at least 28 days (Figure 2A and B).

Our present study further showed that MCP-1 was expressed mainly by CD11b-positive reactive microglia within the hilus after SE. Interestingly, the MCP-1 expressing reactive microglia were localized near the ectopic locations of neuroblasts (Figure 2C). In line with our observation of the linkage between MCP-1 expression and microglia, one recent study in MCP-1-deficient mice has demonstrated a reduction in microglial migration to the site of excitatory neurotransmitter release and a reduction in excitotoxic neuron loss [43]. Taken together, reactive microglia might be involved in seizure-induced dentate MCP-1 upregulation and aberrant migration of neuronal progenitors. Moreover, MCP-1 expression was increased in neurons and astrocytes in SE rats of the present study. We have previously proposed that the upregulated MCP-1 expression in various cell types might participate in the neuroplastic and neuroinflammatory alterations following SE [17].

### Mechanisms of aberrant migration of neural progenitors

In the present study, Dcx-positive neuroblasts in the SGZ expressed the MCP-1 receptor, CCR2, in both control and SE rats, and ectopic Dcx/CCR2-positive cells in the hila were found exclusively in SE rats (Figure 3). The findings suggest that MCP-1 upregulation in the DG might be associated with aberrant migration of neural progenitors. Furthermore, the attenuating effect of a CCR2 antagonist (RS102895) on the formation of hilar-ectopic Prox1/Dcx-positive cells (Figures 5 and 6) suggests an interactive involvement of both MCP-1 and CCR2 in the ectopic migration of neural progenitors toward the hilar region.

However, Prox1/Dcx-expressing neuronal progenitors were still found in the hila of SE rats when CCR2 was inhibited (Figure 5). The migration of neural progenitors in SE rats may be related to several factors. For example, the chemokine stromal cell-derived factor-1α (SDF-1α/CXCL12) has been reported to regulate the migration of dentate granule cells [45] and embryonic stem cell-derived neuronal progenitors through the CXCR4 receptor in the hippocampus [46]. One recent study has further identified a co-expression of CXCR4 and CCR2 in neurogenic cells of mouse brain [45]. Increased expression of SDF-1α/CXCL12 following seizures may influence the migration and/or differentiation of neural progenitors during embryogenesis and in the adult hippocampus [46]. Moreover, Reelin has been reported to modulate neural progenitor migration in rodent and human hippocampal DG. Loss of Reelin might contribute to ectopic chain migration and to aberrant integration of newborn neurons in the epileptic adult hippocampus [41].

## Conclusions

In conclusion, the MCP-1/CCR2 chemokine-receptor interaction may lead to aberrant migration of neuronal progenitors and subsequently contribute to abnormal synaptic integration and epileptogenesis. Further studies are needed to clarify the underlying signaling pathways of aberrant neuronal progenitor migration affected by MCP-1 following SE. This study supports the involvement of neuroinflammation in epileptogenesis.

## Abbreviations

CCR2: Chemokine (C-C motif) receptor 2; Dcx: Doublecortin; DG: Dentate gyrus; ELISA: Enzyme-linked immunosorbent assay; MCP-1: Monocyte chemoattractant protein-1; MTLE: Mesial temporal lobe epilepsy; Prox1: Prospero homeobox protein 1; qRT-PCR: Quantitative real-time reverse transcriptase PCR; SE: Status epilepticus; SGZ: Subgranular zone.

## Competing interests

The authors declare that they have no competing interests.

## Authors’ contributions

YWH contributed to the design and performance of experiments, acquisition and analysis of data, preparing figures, and drafting the manuscript. MTL, YJT and CCC helped to perform experiments, collect qRT-PCR and immunohistochemistry data, and prepare the manuscript. YYL conceived the study, participated in its design and coordination, was involved in drafting the manuscript and revising it critically for important intellectual content. All authors have read and approved the final version of the manuscript.
